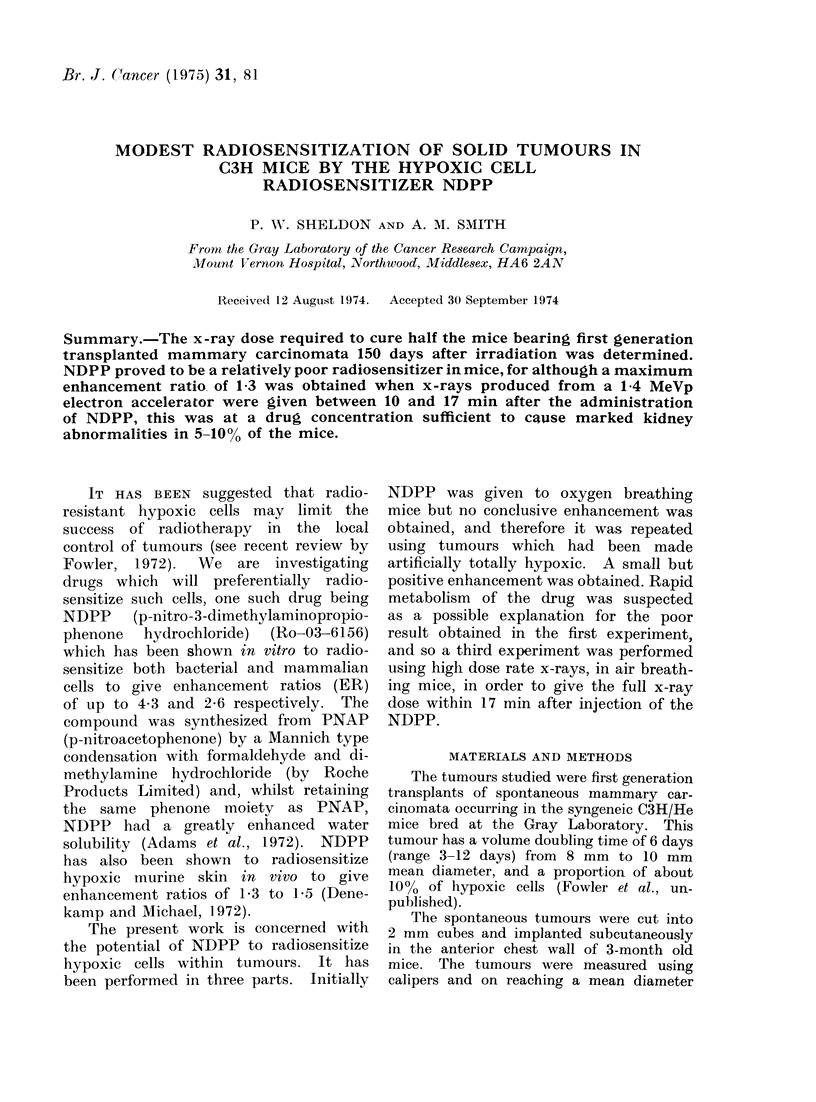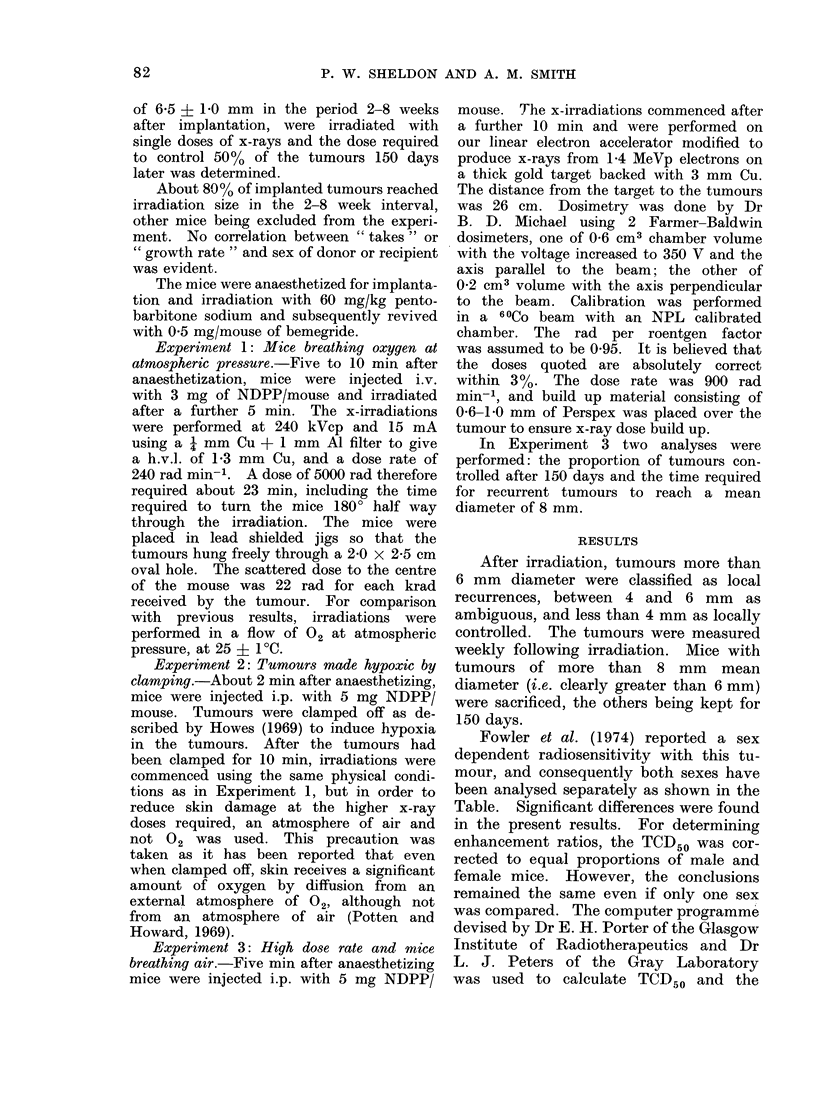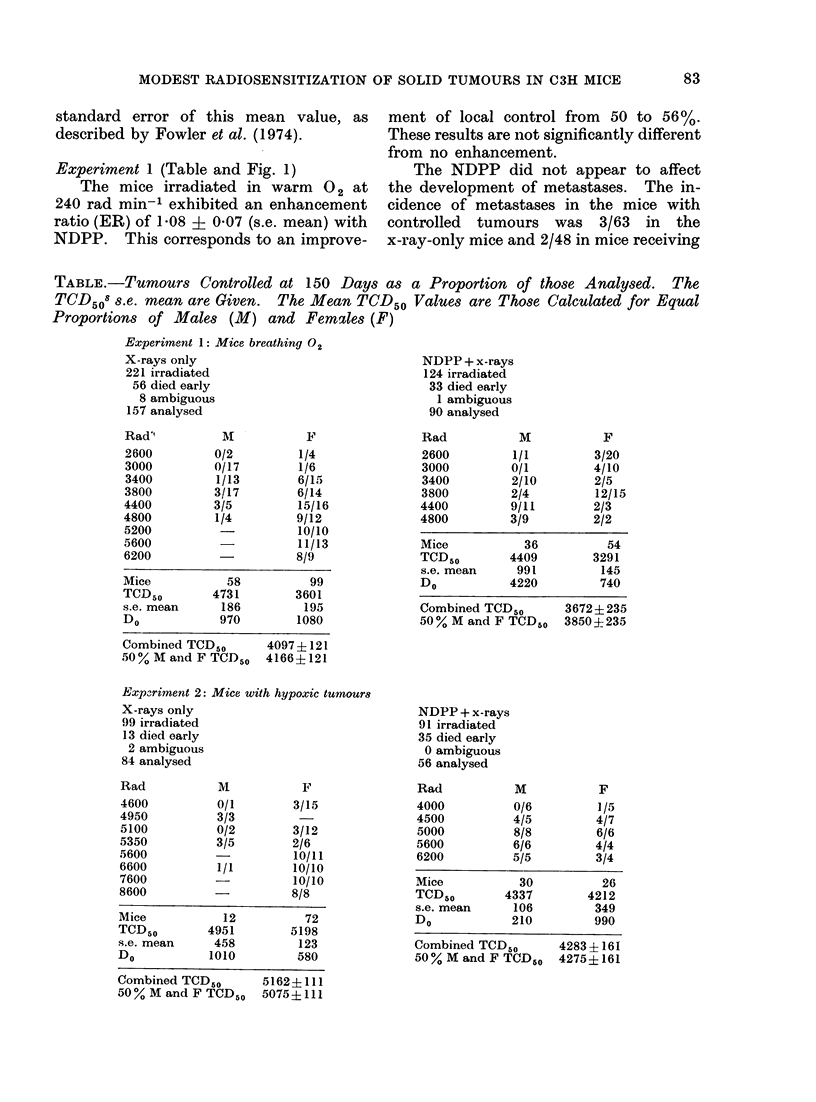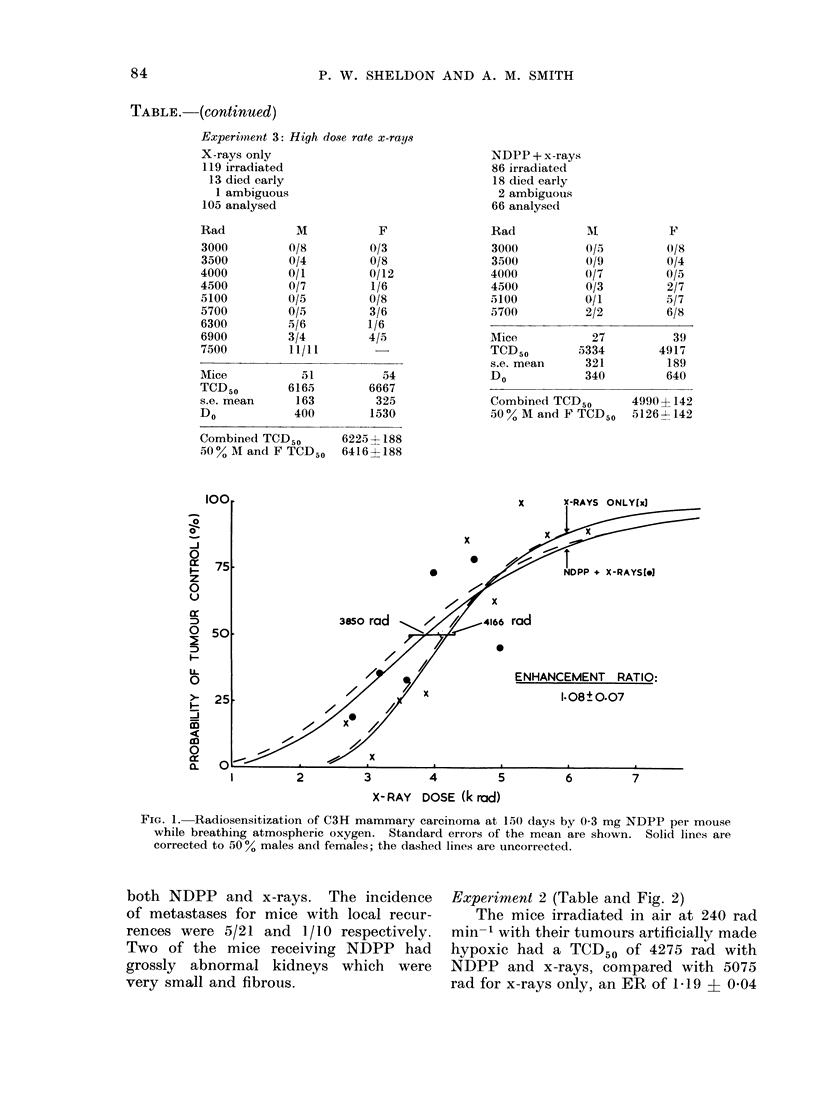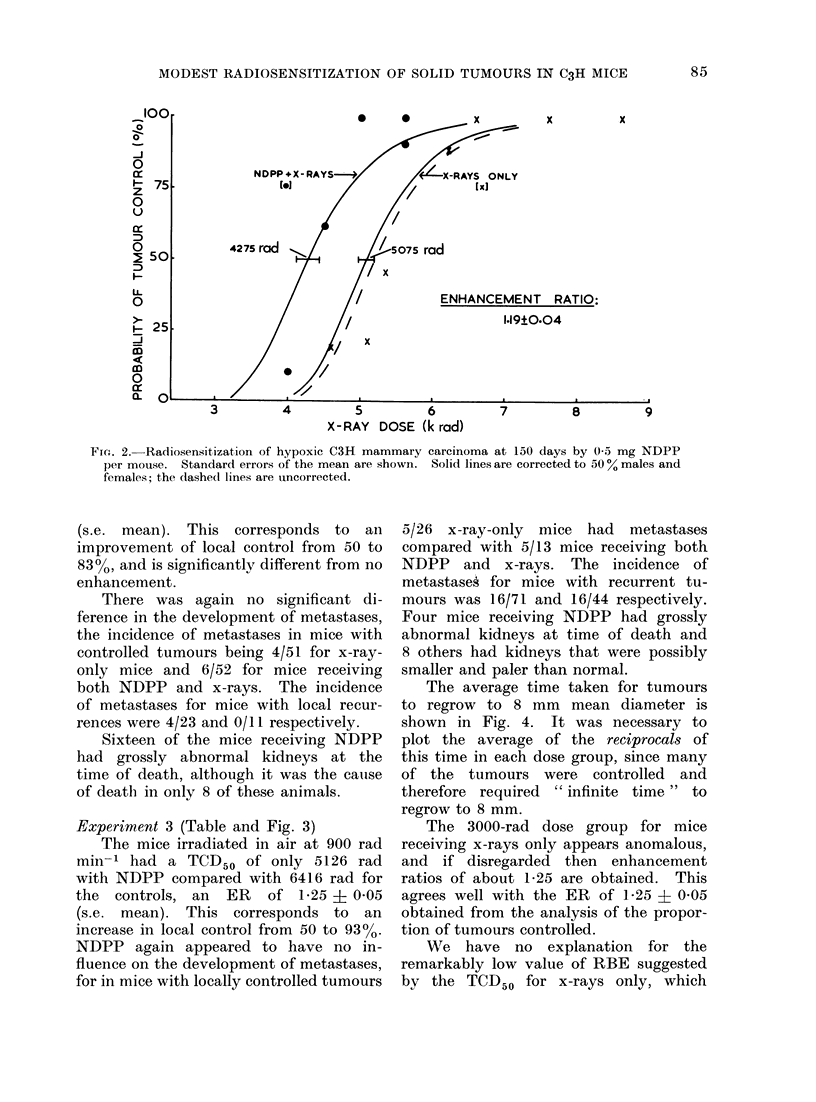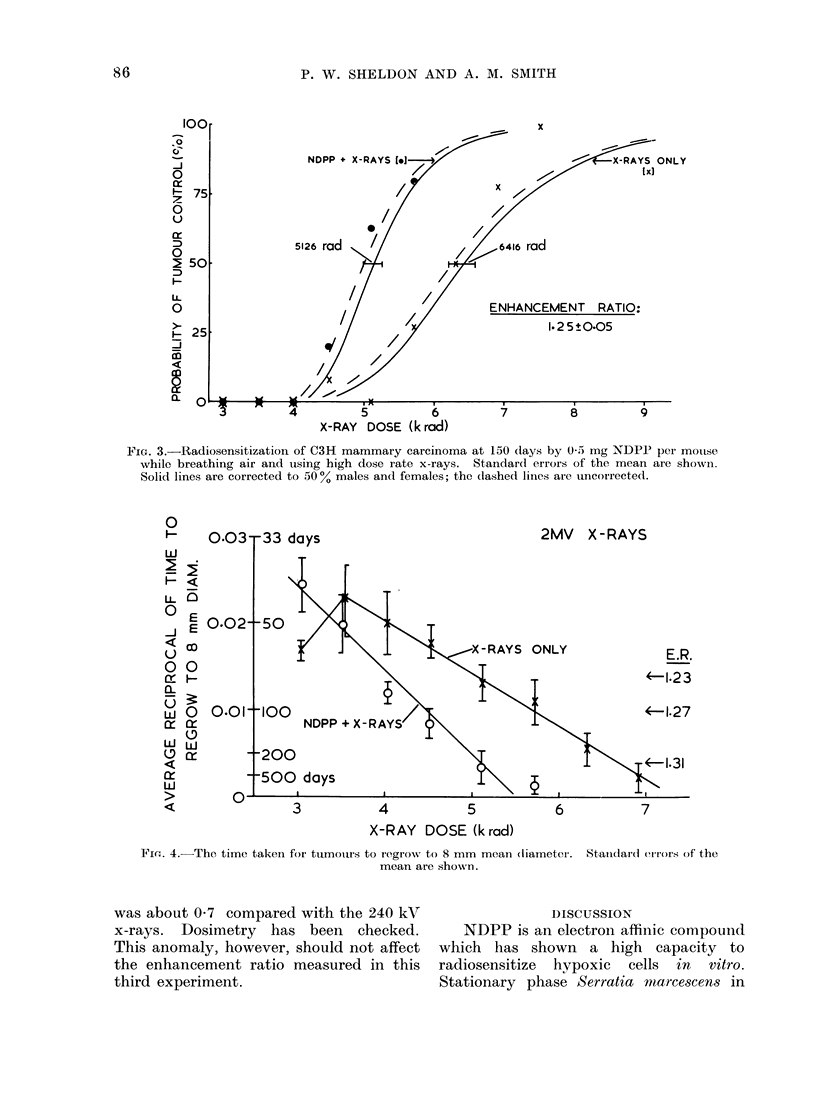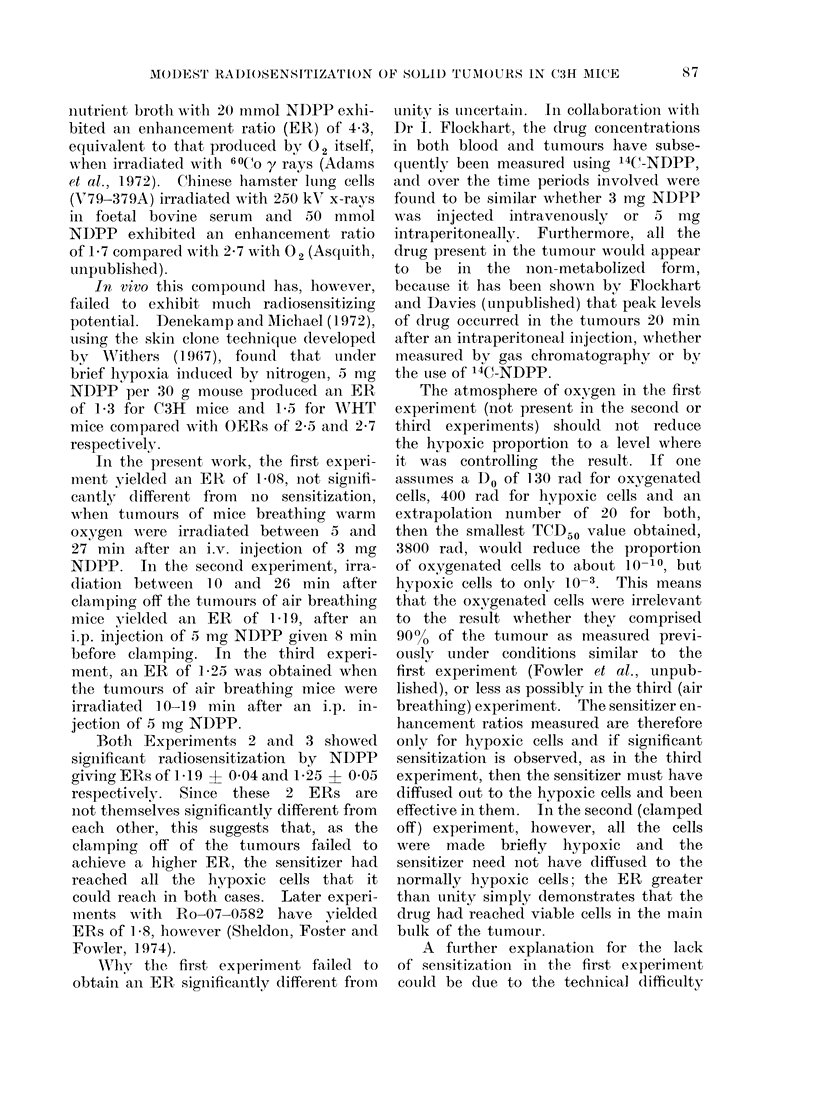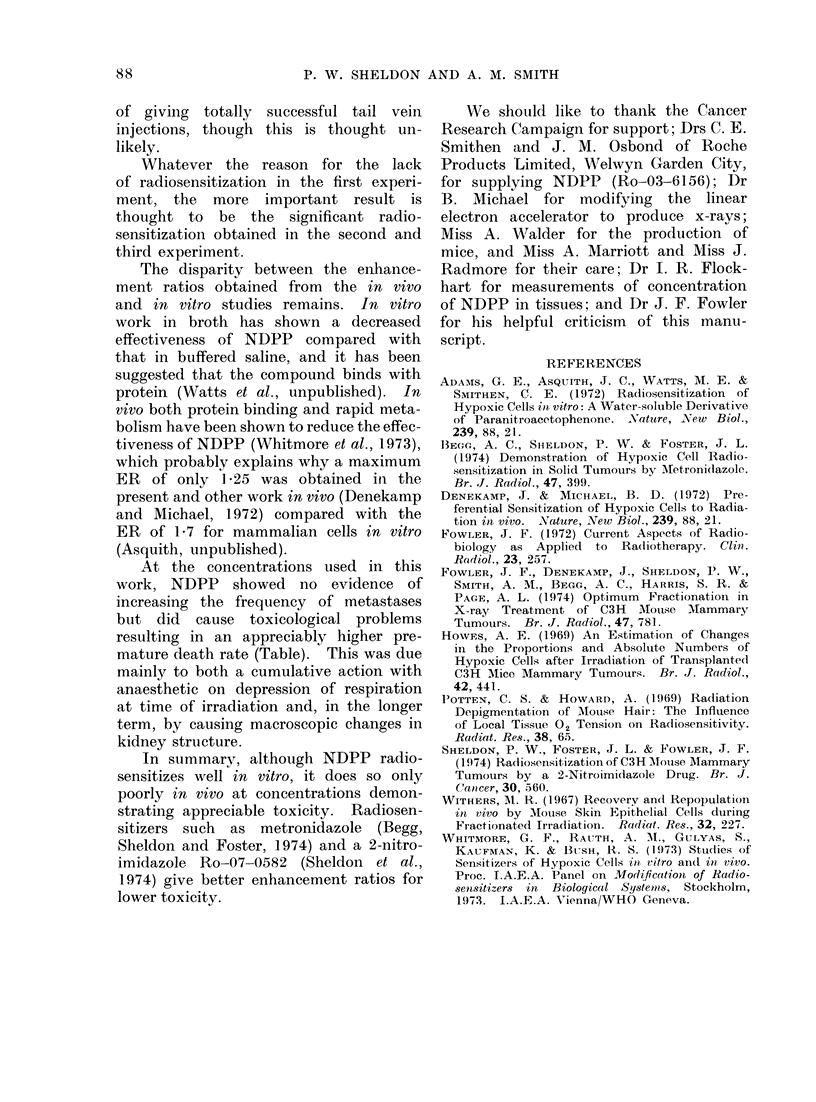# Modest radiosensitization of solid tumours in C3H mice by the hypoxic cell radiosensitizer NDPP.

**DOI:** 10.1038/bjc.1975.10

**Published:** 1975-01

**Authors:** P. W. Sheldon, A. M. Smith

## Abstract

The x-ray dose required to cure half the mice bearing first generation transplanted mammary carcinomata 150 days after irradiation was determined. NDPP proved to be a relatively poor radiosensitizer in mice, for although a maximum enhancement ratio of 1-3 was obtained when x-rays produced from a 1-4 MeVp electron accelerator were given between 10 and 17 min after the administration of NDPP, this was at a drug concentration sufficient to cause marked kidney abnormalities in 5-10% of the mice.


*Br. J. Cancer* (1975) **31**, 81

### MODEST RADIOSENSITIZATION OF SOLID TUMOURS IN C3H MICE BY THE HYPOXIC CELL RADIOSENSITIZER NDPP

P. W. SHELDON AND A. M. SMITH

*From the Gray Laboratory of the Cancer Research Campaign, Mount Vernon Hospital, Northwood, Middlesex, HA6 2AN*

Received 12 August 1974. Accepted 30 September 1974

**Summary.—The x-ray dose required to cure half the mice bearing first generation transplanted mammary carcinomata 150 days after irradiation was determined. NDPP proved to be a relatively poor radiosensitizer in mice, for although a maximum enhancement ratio of 1·3 was obtained when x-rays produced from a 1·4 MeVp electron accelerator were given between 10 and 17 min after the administration of NDPP, this was at a drug concentration sufficient to cause marked kidney abnormalities in 5–10% of the mice.**

IT HAS BEEN suggested that radio-resistant hypoxic cells may limit the success of radiotherapy in the local control of tumours (see recent review by Fowler, 1972). We are investigating drugs which will preferentially radio-sensitize such cells, one such drug being NDPP (p-nitro-3-dimethylaminopropio-phenone hydrochloride) (Ro–03–6156) which has been shown *in vitro* to radio-sensitize both bacterial and mammalian cells to give enhancement ratios (ER) of up to 4·3 and 2·6 respectively. The compound was synthesized from PNAP (p-nitroacetophenone) by a Mannich type condensation with formaldehyde and di-methylamine hydrochloride (by Roche Products Limited) and, whilst retaining the same phenone moiety as PNAP, NDPP had a greatly enhanced water solubility (Adams *et al.*, 1972). NDPP has also been shown to radiosensitize hypoxic murine skin *in vivo* to give enhancement ratios of 1·3 to 1·5 (Dene-kamp and Michael, 1972).

The present work is concerned with the potential of NDPP to radiosensitize hypoxic cells within tumours. It has been performed in three parts. Initially NDPP was given to oxygen breathing mice but no conclusive enhancement was obtained, and therefore it was repeated using tumours which had been made artificially totally hypoxic. A small but positive enhancement was obtained. Rapid metabolism of the drug was suspected as a possible explanation for the poor result obtained in the first experiment, and so a third experiment was performed using high dose rate x-rays, in air breath-ing mice, in order to give the full x-ray dose within 17 min after injection of the NDPP.

### MATERIALS AND METHODS

The tumours studied were first generation transplants of spontaneous mammary car-cinomata occurring in the syngeneic C3H/He mice bred at the Gray Laboratory. This tumour has a volume doubling time of 6 days (range 3–12 days) from 8 mm to 10 mm mean diameter, and a proportion of about 10% of hypoxic cells (Fowler *et al.*, un-published).

The spontaneous tumours were cut into 2 mm cubes and implanted subcutaneously in the anterior chest wall of 3-month old mice. The tumours were measured using calipers and on reaching a mean diameter of $6 \cdot 5 \pm 1 \cdot 0$ mm in the period 2–8 weeks after implantation, were irradiated with single doses of x-rays and the dose required to control 50% of the tumours 150 days later was determined.

About 80% of implanted tumours reached irradiation size in the 2–8 week interval, other mice being excluded from the experiment. No correlation between " takes " or " growth rate " and sex of donor or recipient was evident.

The mice were anaesthetized for implantation and irradiation with 60 mg/kg pentobarbitone sodium and subsequently revived with 0·5 mg/mouse of bemegride.

*Experiment* 1: *Mice breathing oxygen at atmospheric pressure.*—Five to 10 min after anaesthetization, mice were injected i.v. with 3 mg of NDPP/mouse and irradiated after a further 5 min. The x-irradiations were performed at 240 kVcp and 15 mA using a $\frac{1}{4}$ mm Cu + 1 mm Al filter to give a h.v.l. of 1·3 mm Cu, and a dose rate of 240 rad $min^{-1}$. A dose of 5000 rad therefore required about 23 min, including the time required to turn the mice 180° half way through the irradiation. The mice were placed in lead shielded jigs so that the tumours hung freely through a $2 \cdot 0 \times 2 \cdot 5$ cm oval hole. The scattered dose to the centre of the mouse was 22 rad for each krad received by the tumour. For comparison with previous results, irradiations were performed in a flow of $O_2$ at atmospheric pressure, at 25 ± 1°C.

*Experiment* 2: *Tumours made hypoxic by clamping.*—About 2 min after anaesthetizing, mice were injected i.p. with 5 mg NDPP/mouse. Tumours were clamped off as described by Howes (1969) to induce hypoxia in the tumours. After the tumours had been clamped for 10 min, irradiations were commenced using the same physical conditions as in Experiment 1, but in order to reduce skin damage at the higher x-ray doses required, an atmosphere of air and not $O_2$ was used. This precaution was taken as it has been reported that even when clamped off, skin receives a significant amount of oxygen by diffusion from an external atmosphere of $O_2$, although not from an atmosphere of air (Potten and Howard, 1969).

*Experiment* 3: *High dose rate and mice breathing air.*—Five min after anaesthetizing mice were injected i.p. with 5 mg NDPP/mouse. The x-irradiations commenced after a further 10 min and were performed on our linear electron accelerator modified to produce x-rays from 1·4 MeVp electrons on a thick gold target backed with 3 mm Cu. The distance from the target to the tumours was 26 cm. Dosimetry was done by Dr B. D. Michael using 2 Farmer–Baldwin dosimeters, one of 0·6 $cm^3$ chamber volume with the voltage increased to 350 V and the axis parallel to the beam; the other of 0·2 $cm^3$ volume with the axis perpendicular to the beam. Calibration was performed in a $^{60}Co$ beam with an NPL calibrated chamber. The rad per roentgen factor was assumed to be 0·95. It is believed that the doses quoted are absolutely correct within 3%. The dose rate was 900 rad $min^{-1}$, and build up material consisting of 0·6–1·0 mm of Perspex was placed over the tumour to ensure x-ray dose build up.

In Experiment 3 two analyses were performed: the proportion of tumours controlled after 150 days and the time required for recurrent tumours to reach a mean diameter of 8 mm.

### RESULTS

After irradiation, tumours more than 6 mm diameter were classified as local recurrences, between 4 and 6 mm as ambiguous, and less than 4 mm as locally controlled. The tumours were measured weekly following irradiation. Mice with tumours of more than 8 mm mean diameter (*i.e.* clearly greater than 6 mm) were sacrificed, the others being kept for 150 days.

Fowler *et al.* (1974) reported a sex dependent radiosensitivity with this tumour, and consequently both sexes have been analysed separately as shown in the Table. Significant differences were found in the present results. For determining enhancement ratios, the $TCD_{50}$ was corrected to equal proportions of male and female mice. However, the conclusions remained the same even if only one sex was compared. The computer programme devised by Dr E. H. Porter of the Glasgow Institute of Radiotherapeutics and Dr L. J. Peters of the Gray Laboratory was used to calculate $TCD_{50}$ and the standard error of this mean value, as described by Fowler *et al.* (1974).

*Experiment* 1 (Table and Fig. 1)

The mice irradiated in warm $O_2$ at 240 rad $min^{-1}$ exhibited an enhancement ratio (ER) of 1·08 ± 0·07 (s.e. mean) with NDPP. This corresponds to an improvement of local control from 50 to 56%. These results are not significantly different from no enhancement.

The NDPP did not appear to affect the development of metastases. The incidence of metastases in the mice with controlled tumours was 3/63 in the x-ray-only mice and 2/48 in mice receiving

TABLE.—*Tumours Controlled at* 150 *Days as a Proportion of those Analysed. The* $TCD_{50}$*s s.e. mean are Given. The Mean* $TCD_{50}$ *Values are Those Calculated for Equal Proportions of Males* (*M*) *and Females* (*F*)

*Experiment* 1: *Mice breathing* $O_2$

X-rays only
221 irradiated
56 died early
8 ambiguous
157 analysed

| Rad | M | F |
|---|---|---|
| 2600 | 0/2 | 1/4 |
| 3000 | 0/17 | 1/6 |
| 3400 | 1/13 | 6/15 |
| 3800 | 3/17 | 6/14 |
| 4400 | 3/5 | 15/16 |
| 4800 | 1/4 | 9/12 |
| 5200 | — | 10/10 |
| 5600 | — | 11/13 |
| 6200 | — | 8/9 |
| Mice | 58 | 99 |
| $TCD_{50}$ | 4731 | 3601 |
| s.e. mean | 186 | 195 |
| $D_0$ | 970 | 1080 |
| Combined $TCD_{50}$ | | 4097 ± 121 |
| 50% M and F $TCD_{50}$ | | 4166 ± 121 |

NDPP + x-rays
124 irradiated
33 died early
1 ambiguous
90 analysed

| Rad | M | F |
|---|---|---|
| 2600 | 1/1 | 3/20 |
| 3000 | 0/1 | 4/10 |
| 3400 | 2/10 | 2/5 |
| 3800 | 2/4 | 12/15 |
| 4400 | 9/11 | 2/3 |
| 4800 | 3/9 | 2/2 |
| Mice | 36 | 54 |
| $TCD_{50}$ | 4409 | 3291 |
| s.e. mean | 991 | 145 |
| $D_0$ | 4220 | 740 |
| Combined $TCD_{50}$ | | 3672 ± 235 |
| 50% M and F $TCD_{50}$ | | 3850 ± 235 |

*Experiment* 2: *Mice with hypoxic tumours*

X-rays only
99 irradiated
13 died early
2 ambiguous
84 analysed

| Rad | M | F |
|---|---|---|
| 4600 | 0/1 | 3/15 |
| 4950 | 3/3 | — |
| 5100 | 0/2 | 3/12 |
| 5350 | 3/5 | 2/6 |
| 5600 | — | 10/11 |
| 6600 | 1/1 | 10/10 |
| 7600 | — | 10/10 |
| 8600 | — | 8/8 |
| Mice | 12 | 72 |
| $TCD_{50}$ | 4951 | 5198 |
| s.e. mean | 458 | 123 |
| $D_0$ | 1010 | 580 |
| Combined $TCD_{50}$ | | 5162 ± 111 |
| 50% M and F $TCD_{50}$ | | 5075 ± 111 |

NDPP + x-rays
91 irradiated
35 died early
0 ambiguous
56 analysed

| Rad | M | F |
|---|---|---|
| 4000 | 0/6 | 1/5 |
| 4500 | 4/5 | 4/7 |
| 5000 | 8/8 | 6/6 |
| 5600 | 6/6 | 4/4 |
| 6200 | 5/5 | 3/4 |
| Mice | 30 | 26 |
| $TCD_{50}$ | 4337 | 4212 |
| s.e. mean | 106 | 349 |
| $D_0$ | 210 | 990 |
| Combined $TCD_{50}$ | | 4283 ± 161 |
| 50% M and F $TCD_{50}$ | | 4275 ± 161 |

TABLE.—(*continued*)

*Experiment* 3: *High dose rate x-rays*

X-rays only
119 irradiated
13 died early
1 ambiguous
105 analysed

| Rad | M | F |
|---|---|---|
| 3000 | 0/8 | 0/3 |
| 3500 | 0/4 | 0/8 |
| 4000 | 0/1 | 0/12 |
| 4500 | 0/7 | 1/6 |
| 5100 | 0/5 | 0/8 |
| 5700 | 0/5 | 3/6 |
| 6300 | 5/6 | 1/6 |
| 6900 | 3/4 | 4/5 |
| 7500 | 11/11 | — |
| Mice | 51 | 54 |
| $TCD_{50}$ | 6165 | 6667 |
| s.e. mean | 163 | 325 |
| $D_0$ | 400 | 1530 |
| Combined $TCD_{50}$ | 6225 ± 188 | |
| 50% M and F $TCD_{50}$ | 6416 ± 188 | |

NDPP + x-rays
86 irradiated
18 died early
2 ambiguous
66 analysed

| Rad | M | F |
|---|---|---|
| 3000 | 0/5 | 0/8 |
| 3500 | 0/9 | 0/4 |
| 4000 | 0/7 | 0/5 |
| 4500 | 0/3 | 2/7 |
| 5100 | 0/1 | 5/7 |
| 5700 | 2/2 | 6/8 |
| Mice | 27 | 39 |
| $TCD_{50}$ | 5334 | 4917 |
| s.e. mean | 321 | 189 |
| $D_0$ | 340 | 640 |
| Combined $TCD_{50}$ | 4990 ± 142 | |
| 50% M and F $TCD_{50}$ | 5126 ± 142 | |

FIG. 1.—Radiosensitization of C3H mammary carcinoma at 150 days by 0·3 mg NDPP per mouse while breathing atmospheric oxygen. Standard errors of the mean are shown. Solid lines are corrected to 50% males and females; the dashed lines are uncorrected.

both NDPP and x-rays. The incidence of metastases for mice with local recurrences were 5/21 and 1/10 respectively. Two of the mice receiving NDPP had grossly abnormal kidneys which were very small and fibrous.

*Experiment* 2 (Table and Fig. 2)

The mice irradiated in air at 240 rad $min^{-1}$ with their tumours artificially made hypoxic had a $TCD_{50}$ of 4275 rad with NDPP and x-rays, compared with 5075 rad for x-rays only, an ER of 1·19 ± 0·04

FIG. 2.—Radiosensitization of hypoxic C3H mammary carcinoma at 150 days by 0·5 mg NDPP per mouse. Standard errors of the mean are shown. Solid lines are corrected to 50 % males and females; the dashed lines are uncorrected.

(s.e. mean). This corresponds to an improvement of local control from 50 to 83%, and is significantly different from no enhancement.

There was again no significant diference in the development of metastases, the incidence of metastases in mice with controlled tumours being 4/51 for x-ray-only mice and 6/52 for mice receiving both NDPP and x-rays. The incidence of metastases for mice with local recurrences were 4/23 and 0/11 respectively.

Sixteen of the mice receiving NDPP had grossly abnormal kidneys at the time of death, although it was the cause of death in only 8 of these animals.

*Experiment* 3 (Table and Fig. 3)

The mice irradiated in air at 900 rad $min^{-1}$ had a $TCD_{50}$ of only 5126 rad with NDPP compared with 6416 rad for the controls, an ER of $1{\cdot}25 \pm 0{\cdot}05$ (s.e. mean). This corresponds to an increase in local control from 50 to 93%. NDPP again appeared to have no influence on the development of metastases, for in mice with locally controlled tumours 5/26 x-ray-only mice had metastases compared with 5/13 mice receiving both NDPP and x-rays. The incidence of metastases for mice with recurrent tumours was 16/71 and 16/44 respectively. Four mice receiving NDPP had grossly abnormal kidneys at time of death and 8 others had kidneys that were possibly smaller and paler than normal.

The average time taken for tumours to regrow to 8 mm mean diameter is shown in Fig. 4. It was necessary to plot the average of the *reciprocals* of this time in each dose group, since many of the tumours were controlled and therefore required " infinite time " to regrow to 8 mm.

The 3000-rad dose group for mice receiving x-rays only appears anomalous, and if disregarded then enhancement ratios of about 1·25 are obtained. This agrees well with the ER of $1{\cdot}25 \pm 0{\cdot}05$ obtained from the analysis of the proportion of tumours controlled.

We have no explanation for the remarkably low value of RBE suggested by the $TCD_{50}$ for x-rays only, which

Fig. 3.—Radiosensitization of C3H mammary carcinoma at 150 days by 0·5 mg NDPP per mouse while breathing air and using high dose rate x-rays. Standard errors of the mean are shown. Solid lines are corrected to 50% males and females; the dashed lines are uncorrected.

Fig. 4.—The time taken for tumours to regrow to 8 mm mean diameter. Standard errors of the mean are shown.

was about 0·7 compared with the 240 kV x-rays. Dosimetry has been checked. This anomaly, however, should not affect the enhancement ratio measured in this third experiment.

### DISCUSSION

NDPP is an electron affinic compound which has shown a high capacity to radiosensitize hypoxic cells *in vitro*. Stationary phase *Serratia marcescens* in nutrient broth with 20 mmol NDPP exhibited an enhancement ratio (ER) of 4·3, equivalent to that produced by $O_2$ itself, when irradiated with $^{60}Co$ $\gamma$ rays (Adams *et al.*, 1972). Chinese hamster lung cells (V79–379A) irradiated with 250 kV x-rays in foetal bovine serum and 50 mmol NDPP exhibited an enhancement ratio of 1·7 compared with 2·7 with $O_2$ (Asquith, unpublished).

*In vivo* this compound has, however, failed to exhibit much radiosensitizing potential. Denekamp and Michael (1972), using the skin clone technique developed by Withers (1967), found that under brief hypoxia induced by nitrogen, 5 mg NDPP per 30 g mouse produced an ER of 1·3 for C3H mice and 1·5 for WHT mice compared with OERs of 2·5 and 2·7 respectively.

In the present work, the first experiment yielded an ER of 1·08, not significantly different from no sensitization, when tumours of mice breathing warm oxygen were irradiated between 5 and 27 min after an i.v. injection of 3 mg NDPP. In the second experiment, irradiation between 10 and 26 min after clamping off the tumours of air breathing mice yielded an ER of 1·19, after an i.p. injection of 5 mg NDPP given 8 min before clamping. In the third experiment, an ER of 1·25 was obtained when the tumours of air breathing mice were irradiated 10–19 min after an i.p. injection of 5 mg NDPP.

Both Experiments 2 and 3 showed significant radiosensitization by NDPP giving ERs of 1·19 $\pm$ 0·04 and 1·25 $\pm$ 0·05 respectively. Since these 2 ERs are not themselves significantly different from each other, this suggests that, as the clamping off of the tumours failed to achieve a higher ER, the sensitizer had reached all the hypoxic cells that it could reach in both cases. Later experiments with Ro–07–0582 have yielded ERs of 1·8, however (Sheldon, Foster and Fowler, 1974).

Why the first experiment failed to obtain an ER significantly different from unity is uncertain. In collaboration with Dr I. Flockhart, the drug concentrations in both blood and tumours have subsequently been measured using $^{14}C$-NDPP, and over the time periods involved were found to be similar whether 3 mg NDPP was injected intravenously or 5 mg intraperitoneally. Furthermore, all the drug present in the tumour would appear to be in the non-metabolized form, because it has been shown by Flockhart and Davies (unpublished) that peak levels of drug occurred in the tumours 20 min after an intraperitoneal injection, whether measured by gas chromatography or by the use of $^{14}C$-NDPP.

The atmosphere of oxygen in the first experiment (not present in the second or third experiments) should not reduce the hypoxic proportion to a level where it was controlling the result. If one assumes a $D_0$ of 130 rad for oxygenated cells, 400 rad for hypoxic cells and an extrapolation number of 20 for both, then the smallest $TCD_{50}$ value obtained, 3800 rad, would reduce the proportion of oxygenated cells to about $10^{-10}$, but hypoxic cells to only $10^{-3}$. This means that the oxygenated cells were irrelevant to the result whether they comprised 90% of the tumour as measured previously under conditions similar to the first experiment (Fowler *et al.*, unpublished), or less as possibly in the third (air breathing) experiment. The sensitizer enhancement ratios measured are therefore only for hypoxic cells and if significant sensitization is observed, as in the third experiment, then the sensitizer must have diffused out to the hypoxic cells and been effective in them. In the second (clamped off) experiment, however, all the cells were made briefly hypoxic and the sensitizer need not have diffused to the normally hypoxic cells; the ER greater than unity simply demonstrates that the drug had reached viable cells in the main bulk of the tumour.

A further explanation for the lack of sensitization in the first experiment could be due to the technical difficulty of giving totally successful tail vein injections, though this is thought unlikely.

Whatever the reason for the lack of radiosensitization in the first experiment, the more important result is thought to be the significant radiosensitization obtained in the second and third experiment.

The disparity between the enhancement ratios obtained from the *in vivo* and *in vitro* studies remains. *In vitro* work in broth has shown a decreased effectiveness of NDPP compared with that in buffered saline, and it has been suggested that the compound binds with protein (Watts *et al.*, unpublished). *In vivo* both protein binding and rapid metabolism have been shown to reduce the effectiveness of NDPP (Whitmore *et al.*, 1973), which probably explains why a maximum ER of only 1·25 was obtained in the present and other work *in vivo* (Denekamp and Michael, 1972) compared with the ER of 1·7 for mammalian cells *in vitro* (Asquith, unpublished).

At the concentrations used in this work, NDPP showed no evidence of increasing the frequency of metastases but did cause toxicological problems resulting in an appreciably higher premature death rate (Table). This was due mainly to both a cumulative action with anaesthetic on depression of respiration at time of irradiation and, in the longer term, by causing macroscopic changes in kidney structure.

In summary, although NDPP radiosensitizes well *in vitro*, it does so only poorly *in vivo* at concentrations demonstrating appreciable toxicity. Radiosensitizers such as metronidazole (Begg, Sheldon and Foster, 1974) and a 2-nitroimidazole Ro–07–0582 (Sheldon *et al.*, 1974) give better enhancement ratios for lower toxicity.

We should like to thank the Cancer Research Campaign for support; Drs C. E. Smithen and J. M. Osbond of Roche Products Limited, Welwyn Garden City, for supplying NDPP (Ro–03–6156); Dr B. Michael for modifying the linear electron accelerator to produce x-rays; Miss A. Walder for the production of mice, and Miss A. Marriott and Miss J. Radmore for their care; Dr I. R. Flockhart for measurements of concentration of NDPP in tissues; and Dr J. F. Fowler for his helpful criticism of this manuscript.

### REFERENCES

Adams, G. E., Asquith, J. C., Watts, M. E. & Smithen, C. E. (1972) Radiosensitization of Hypoxic Cells *in vitro*: A Water-soluble Derivative of Paranitroacetophenone. *Nature, New Biol.*, **239**, 88, 21.

Begg, A. C., Sheldon, P. W. & Foster, J. L. (1974) Demonstration of Hypoxic Cell Radiosensitization in Solid Tumours by Metronidazole. *Br. J. Radiol.*, **47**, 399.

Denekamp, J. & Michael, B. D. (1972) Preferential Sensitization of Hypoxic Cells to Radiation *in vivo*. *Nature, New Biol.*, **239**, 88, 21.

Fowler, J. F. (1972) Current Aspects of Radiobiology as Applied to Radiotherapy. *Clin. Radiol.*, **23**, 257.

Fowler, J. F., Denekamp, J., Sheldon, P. W., Smith, A. M., Begg, A. C., Harris, S. R. & Page, A. L. (1974) Optimum Fractionation in X-ray Treatment of C3H Mouse Mammary Tumours. *Br. J. Radiol.*, **47**, 781.

Howes, A. E. (1969) An Estimation of Changes in the Proportions and Absolute Numbers of Hypoxic Cells after Irradiation of Transplanted C3H Mice Mammary Tumours. *Br. J. Radiol.*, **42**, 441.

Potten, C. S. & Howard, A. (1969) Radiation Depigmentation of Mouse Hair: The Influence of Local Tissue $O_2$ Tension on Radiosensitivity. *Radiat. Res.*, **38**, 65.

Sheldon, P. W., Foster, J. L. & Fowler, J. F. (1974) Radiosensitization of C3H Mouse Mammary Tumours by a 2-Nitroimidazole Drug. *Br. J. Cancer*, **30**, 560.

Withers, M. R. (1967) Recovery and Repopulation *in vivo* by Mouse Skin Epithelial Cells during Fractionated Irradiation. *Radiat. Res.*, **32**, 227.

Whitmore, G. F., Rauth, A. M., Gulyas, S., Kaufman, K. & Bush, R. S. (1973) Studies of Sensitizers of Hypoxic Cells *in vitro* and *in vivo*. Proc. I.A.E.A. Panel on *Modification of Radiosensitizers in Biological Systems*, Stockholm, 1973. I.A.E.A. Vienna/WHO Geneva.